# Supplementary material for: Micropulse cyclophotocoagulation compared to continuous wave cyclophotocoagulation for the management of refractory pediatric glaucoma
Source: PLoS One. 2024 Jan 2;19(1):e0291247. doi: 10.1371/journal.pone.0291247 (PMC10760766; doi:10.1371/journal.pone.0291247)
Supplement: S1 File — (PDF) [file pone.0291247.s001.pdf]

Procedure	Power	Duration	Shots	Eye	age at surger	Previous Surg
TSCPC	1250		32	OS	14	0
TSCPC	2000	2	17	OS	3	3
TSCPC	1125	4	13	OD	0	0
TSCPC	1800	2	10	OD	1	0
TSCPC	1700	2	12	OS	1	0
TSCPC	1900	3	23	OD	18	0
TSCPC	1125	4	20	OS	7	0
TSCPC	1600	4	20	OS	9	1
TSCPC	700	4	20	OD	17	0
TSCPC	1250	4	16	OD	10	2
TSCPC	2150	2	14	OS	6	1
TSCPC	1050	4	19	OD	7	3
TSCPC	1500	2	17	OS	1	1
TSCPC	1200		10	OS	16	2
TSCPC	1500		10	OD	17	1
TSCPC	1650	4	24	OD	12	5
TSCPC	2000	2	15	OD	2	1
TSCPC	2000	2	15	OS	2	2
TSCPC	1600	2	15	OD	6	1
TSCPC	1250		16	OS	7	2
TSCPC	2000	2	15	OD	16	1
TSCPC	1250	4	18	OD	8	4
TSCPC	1250	4	18	OD	8	4
TSCPC	2010		9	OS	17	0
TSCPC	1075	4	24	OS	6	0
TSCPC	2000		20	OS	14	2
TSCPC	1400	4	23	OD	10	0
TSCPC	2000	2	17	OS	10	0
TSCPC	1500	2	16	od	7	3
TSCPC	1500	4	20	OD	13	0
MPCPC	2000	40		OD	5	2
MPCPC	1010	90		OS	2	2
MPCPC	2000	90		OD	1	1
MPCPC	2000	90		OS	13	1
MPCPC	2000	90		OS	12	1
MPCPC	2000	90		OD	10	1
MPCPC	1500	90		OS	7	1
MPCPC	1050	90		OS	2	1
MPCPC	1050	90		OD	2	1
MPCPC	1050	90		OD	1	2
MPCPC	1050	90		OS	1	2
MPCPC	1050	90		OD	4	0

MPCPC	1000	40	OD	1	2
MPCPC	2000	90	OS	7	2
MPCPC	2100	270	OD	8	2
MPCPC	2250	270	OS	14	1
MPCPC	2000	270	OD	9	2
MPCPC	2250	270	OS	11	1
MPCPC	2250	270	OD	12	1
MPCPC	2500	200	OS	10	0
MPCPC	2000	360	OS	10	1
MPCPC	2000	270	OD	7	1
MPCPC	2500	200	OS	12	2
MPCPC	2500	230	OS	9	2
MPCPC	2500	270	OD	9	2
MPCPC	2250	140	OD	1	4

Diamox preo	Preop Meds (	Preop IOP	POM1 IOP	POM1 PF (nu	POM1 Meds	Time to Failu
no	4	41	34	0	4	38
no	2	35	33		2	365
no	2	16	26	2	4	74
no	3	30				227
no	3	37				365
no	0	54	14	2	1	103
no	3	30				365
no	3	40				89
no	4	35	51	0	4	12
no	1	23	11		0	365
yes	4	14	10	2	2	365
yes	4	38				43
yes	3	37			0	91
no	4	27	21	0	2	365
no	4	28	7	0	0	365
no	4	28	17	2	2	365
no	3	22				118
no	3	33	18			182
no	0	14	23	0	4	83
no	3	16	16	2	3	365
no	0	10	20	2	0	365
no	4	44	26	0	4	85
no	4	44	26	0	3	85
yes	4	37	32			365
no	2	48				365
no	2	35	26	4	2	85
no	2	45	28	4	2	237
yes	4	40	24	4	4	62
no	3	35	23	4	2	52
no	1	36	11	4	0	365
no	4	37	28	4	0	28
no	3	37	36			9
no	2	25	14	1	0	365
no	4	39	31	0	2	6
no	4	30				114
no	1	36	38	4		30
no	3	32	29	0	3	10
no	3	31	32	2	2	9
no	3	15				7
no	3	15	17	4	2	365
no	0	14	15	2	2	365
no	0	31	45		0	31

no	3	31	24		3	62
no	2	31	13	0	2	1
no	3	25	20	0	3	182
no	3	31	13	0	3	365
no	3	35	18	0	2	365
no	3	28	29	0	3	30
no	3	23	19	0	3	365
yes	3	38	16	0	2	10
no	4	37	18	0	4	91
no	3	34	19	0	3	91
no	4	18				365
no	3	33	35	0	3	30
yes	3	34	6	0	3	37
no	2	6	21	2	3	35

ıre
