# Supplementary material for: Micropulse cyclophotocoagulation compared to continuous wave cyclophotocoagulation for the management of refractory pediatric glaucoma
Source: PLoS One. 2024 Jan 2;19(1):e0291247. doi: 10.1371/journal.pone.0291247 (PMC10760766; doi:10.1371/journal.pone.0291247)
Supplement: Attachment — Submitted filename: Review Comments.pdf [file pone.0291247.s002.pdf]

We thank both reviewers for their excellent comments and feedback. Please find below a point-by-point response of their comments below. During data upload, it was noted that 2 patients in the TSCPC group who were excluded during the data analysis (insufficient follow up) were accidentally counted in Table 1. The table and abstract were updated to reflect that exclusion and there is no change to the results or conclusion of the paper.

Reviewer 1:
The manscript "Micropulse Cyclophotocoagulation Compared to Continuous Wave Cyclophotocoagulation for the Management of Refractory Pediatric Glaucoma" is compiled to take in view to manage the pediatric glaucoma. over all, the manuscript conclude that MPCPC has short term effect on IOP lowering and CWCPC is more reliable than MPCPC.
I have only one question about this study. How would you justify your comparative study with a previous study as mentioned below
Micropulse versus continuous wave transscleral diode cyclophotocoagulation in refractory glaucoma: a randomized exploratory study in clinical & experimental ophthalmology 2015.
what do you think, which augmentation in MPCPC will become fruitful for the future studies.

We completely agree that augmentation in MPCPC could potentially improve the capability of MPCPC to lower IOP. Future work is required to evaluate these enhanced settings. “Likewise, trials with standardized power settings, such as those recently advocated by Grippo et al. in their consensus guidelines, may result in optimized settings for pediatric patients.[17]”

The published study shows extraordinary results of MPCPC compared to continuous wave laser, with power settings like ours. There are several differences that can account for this, the authors allowed retreatment (half the patients received retreatment) and the study was performed in adults, who might respond differently than children to micropulse. We added: “Adult studies comparing MPCPC to CWCPC show excellent results with MPCPC, however, they also allowed retreatment and these findings may reflect differences in how adult and children respond to treatment.”

Reviewer 2:
Manuscript is technically sound and data support conclusion. Data is available according to findings mentioned in manuscript but there is need to write about written consent of glaucoma patients about treatment in methods section.

We added the following to the methods section: “The IRB at both institutions waived the need for signed consent for this retrospective review and the data was anonymized at each institution.”
