# Supplementary material for: Micropulse cyclophotocoagulation compared to continuous wave cyclophotocoagulation for the management of refractory pediatric glaucoma
Source: PLoS One. 2024 Jan 2;19(1):e0291247. doi: 10.1371/journal.pone.0291247 (PMC10760766; doi:10.1371/journal.pone.0291247)
Supplement: Attachment — Submitted filename: Micropulse Cyclophotocoagulation to Continuous Wave.pdf [file pone.0291247.s003.pdf]

Comments to authors:

The manuscript deals with an important topic and covers an area of research not sufficiently covered in previous research. The authors conducted a retrospective study to compare "Micropulse Cyclophotocoagulation to Continuous Wave Cyclophotocoagulation for the Management of Refractory Pediatric Glaucoma" The manuscript concludes that in pediatric glaucoma patients undergoing cyclophotocoagulation procedures, CWCPC outperformed MPCPC using default settings in terms of achieving IOP control for at least one year.

Overall; the paper is well-written and organized. Here below; some comments:

Introduction

This section contains enough general and specific background information but we need more description of the gap in our knowledge that the study was designed to fill.

Methodology

1)-The authors have to mention some details about the instruments used in the clinical examination of the patients, especially the IOP measuring device and whether IOP measurements were done under general anaesthesia or not, and what type of anaesthesia used

2)-The authors have to mention some details about how the Cyclophotocoagulation probe was applied in both groups e.g

MP-CPC Group:

- The probe was applied with firm pressure, perpendicular to the sclera, with the probe edge ……etc
- Avoiding the 3 and 9 o'clock meridians?

CW-CPC Group

- Transillumination with a light source in a darkened theater to accurately locate the position of the ciliary body was used or not?
- The power and duration of the laser application were increased until a popping sound was heard. Is this method followed or not?
- The number of popping shots?
- Avoiding 3 and 9 o'clock positions

Discussion

This section was more or less well-written with a clear comparison to the previously published results, however, it was missing a detailed comparison with the study of Abdelrahman & El Sayed (16) which is the nearest to the subject of the present study.

MP-CPC Group

The probe was applied with a firm pressure, perpendicular to the sclera, with the edge of the probe directly on the limbus at all times. The probe was moved in a continuous sliding motion in the superior and inferior quadrants, avoiding the 3 and 9 o'clock meridians.

CW-CPC Group

Transillumination with a light source in a darkened theater was used to accurately locate the position of the ciliary body, and the concave side of the footplate was then aligned with the anterior edge of the ciliary body   The power and duration of the laser application were increased until a popping sound was heard. The number of popping shots was left at the discretion of the treating physician. Approximately 15 shots were delivered in the superior quadrants and 15 in the inferior quadrants, avoiding 3 and 9 o'clock positions.
