# Supplementary material for: Micropulse cyclophotocoagulation compared to continuous wave cyclophotocoagulation for the management of refractory pediatric glaucoma
Source: PLoS One. 2024 Jan 2;19(1):e0291247. doi: 10.1371/journal.pone.0291247 (PMC10760766; doi:10.1371/journal.pone.0291247)
Supplement: Attachment — Submitted filename: Response to reviewers 2.docx [file pone.0291247.s004.docx]

We would like to thank both reviewers for their insightful comments regarding the manuscript. We feel that the manuscript has been substantially improved as a result, and hope that it will now be acceptable for publication in *PLOS ONE*. Please find below a point-by-point response to the feedback from the Reviewers.

Comments to authors:

The manuscript deals with an important topic and covers an area of research not sufficiently covered in previous research. The authors conducted a retrospective study to compare "Micropulse Cyclophotocoagulation to Continuous Wave Cyclophotocoagulation for the Management of Refractory Pediatric Glaucoma" The manuscript concludes that in pediatric glaucoma patients undergoing cyclophotocoagulation procedures, CWCPC outperformed MPCPC using default settings in terms of achieving IOP control for at least one year.

Overall; the paper is well-written and organized. Here below; some comments:

Introduction

This section contains enough general and specific background information but we need more description of the gap in our knowledge that the study was designed to fill.

Methodology

1)-The authors have to mention some details about the instruments used in the clinical examination of the patients, especially the IOP measuring device and whether IOP measurements were done under general anaesthesia or not, and what type of anaesthesia used.

As this was a retrospective study across two different institutions. IOP measurement were done based on the preference of the institution and not standardized. However, the trend of improvement or lack thereof should not be significantly impacted on this.

We added: “All patients underwent general anesthesia for the procedure.”

2)-The authors have to mention some details about how the Cyclophotocoagulation probe was applied in both groups e.g

MP-CPC Group:

• The probe was applied with firm pressure, perpendicular to the sclera, with the probe edge ......etc

• Avoiding the 3 and 9 o’clock meridians?

We added: “MPCPC probe was firmly applied at the location of ciliary body and painted circumferentially at the limbus, with care taken to avoid the 3 and 9 o’clock meridian. When the limbus was challenging to identify, transillumination was used to assist in probe positioning.”

CW-CPC Group

• Transillumination with a light source in a darkened theater to accurately locate the position of the ciliary body was used or not?

• The power and duration of the laser application were increased until a popping sound was heard. Is this method followed or not?

• The number of popping shots?

• Avoiding 3 and 9 o’clock positions

Unfortunately, due to this being a retrospective study across two institutions, there was not a standardized method performed to located the ciliary body. The number of popping shots vs non-popping shots were not recorded in the charts.

We added: “The CWCPC probe was also firmly applied at the location of the ciliary body and individual applications were applied circumferentially at the limbus with care to avoid the 3 and 9 o’clock position…The power and duration of the CWCPC was titrated down according to popping sounds.”

Discussion

This section was more or less well-written with a clear comparison to the previously published results, however, it was missing a detailed comparison with the study of Abdelrahman & El Sayed (16) which is the nearest to the subject of the present study.

We added: “There are variations reported in the literature regarding the success of MPCPC in children… Abdelrahman et al reports an approximate 70% success rate with similar performance between MPCPC and CWCPC, with the CWCPC showing a worse side effect profile.”
